# Error‐Tolerant Multimodal Vision‐Language Models for Endodontic Triaging: A Cross‐Sectional Study

**DOI:** 10.1155/ijod/4148741

**Published:** 2026-01-31

**Authors:** Md Fahim Shahoriar Titu, Mahir Afser Pavel, Afifa Zain Apurba, Saif Ahmed, Shafin Rahman, James Dudley, Taseef Hasan Farook

**Affiliations:** ^1^ Department of Electrical and Computer Engineering, North South University, Dhaka, Bangladesh, northsouth.edu; ^2^ Adelaide Dental School, University of Adelaide, School of Dentistry, Adelaide University, Adelaide, 5000, South Australia, Australia, adelaide.edu.au

**Keywords:** intraoral radiographs, linguistic metrics, orthopantomogram, quantisation-aware training, root canal treatment

## Abstract

**Objective:**

To assess the performance of multimodal vision‐language artificial intelligence models, optimised using quantisation‐aware training, in triaging endodontic treatment needs. The focus is on the ability to interpret endodontic radiographs while tolerating common image capture errors, including cone cutting, elongation, foreshortening, horizontal misalignment, over‐ and under‐exposure and artefacts.

**Methods:**

In total 3600 dental radiographs were obtained across a 1‐year period. Image augmentation techniques were applied to enhance model generalisability. Bootstrapped Language‐Image Pretraining (BLIP), CLIP, Florence 2 and Paligemma multimodal models were fine‐tuned using quantisation‐aware training and evaluated using Bilingual Evaluation Understudy (BLEU), Recall‐Oriented Understudy for Gisting Evaluation (ROUGE), Metric for Evaluation of Translation with Explicit ORdering (METEOR), Consensus‐based Image Description Evaluation (CIDEr) and Loss Trends and Convergence.

**Results:**

Quantisation‐aware optimisation improved BLEU‐4 by at least 17.3%, METEOR by at least 11.1%, ROUGE‐L by at least 9.8% and CIDEr by at least 75.5% across all models. Quantisation reduced memory consumption by 87.5% while preserving diagnostic accuracy within a 0.5% error margin while correctly reproducing over 90% endodontic triage assessments made by practitioners.

**Conclusion:**

Multimodal AI demonstrates tolerance to imaging inconsistencies and is capable of accurately triaging endodontic cases with minimal computational demands, without compromising diagnostic performance.

## 1. Introduction

Endodontic treatment, commonly known as root canal therapy, involves the removal of diseased, traumatised or infected dental pulp (the soft tissue consisting of nerves and blood vessels) from within a tooth followed by cleaning, shaping, and sealing to promote healing and prevent reinfection  [1]. Endodontic therapy is typically performed in three stages as follows:

1. Initial pulp extirpation involving removing the pulp tissue from the pulp chamber and root canals, then placing an antimicrobial agent within the root canals. The first phase of treatment is effective in relieving pain and is routinely performed in public hospitals as part of emergency dental care.
2. Cleaning, shaping and further disinfecting the root canals.
3. Obturation, or sealing, the root canals.

The first stage of treatment is typically provided in emergency settings. The remaining stages are staged and often referred to private dental clinics and require serial radiographs to verify the length of the root canals, monitor infection resolution, and assess the completeness of the root canal filling. Due to the complexity and cost, patients in public systems frequently delay completing treatment beyond the emergency stage, often leading to reinfection of the tooth.

In public hospitals with dental departments, emergency dental care is delivered to large numbers of patients within strict time constraints. Endodontic triage systems typically classify teeth as requiring treatment, incompletely treated (having undergone initial pulp extirpation only) or fully treated (obturated tooth) using a combination of clinical assessment and radiographic evaluation. However, radiographs taken in these settings often suffer from common acquisition and processing errors such as cone cutting, elongation, foreshortening, horizontal angulation errors, over‐/under‐exposure and image artefacts. This is likely due to time pressures and reliance on less experienced student operators or new graduates [2]. The limitations hinder accurate diagnosis and efficient triage.

### 1.1. Multimodal Vision‐Language Models (VLMs) in Endodontics

Deep learning now provides new opportunities to support diagnostic triage by identifying and segmenting teeth based on endodontic treatment status using both intraoral radiographs and low‐resolution panoramic images, even when the images are compromised by acquisition or processing errors. Traditional convolutional neural networks (CNNs) have identified periapical changes in endodontically treated teeth at commendable accuracies ranging from 89%  [3] to 92%  [4] with some algorithms being able to delineate dental tissue boundaries from cone‐beam computed tomography (CBCT) images  [5].

However, CNNs are limited mostly by their reliance on hierarchical data structures [6]. In contrast, endodontic triage is multidimensional and often complicated by variability in imaging modalities that affect diagnostic accuracy. In contrast, multimodal VLMs are able to consider both global image features and associated textual data to offer a more holistic approach. For instance, Ji et al.  [7] developed Clinical Bootstrapped Language‐Image Pretraining (BLIP), a VLM for radiology report generation, which outperformed traditional network models by achieving a 24% improvement in clinical relevance.

Nevertheless, current VLM implementations still struggle with poor image quality, with many datasets excluding substantial sums of radiographs with acquisition errors, and existing approaches often prioritise object detection over layered clinical decision support.

### 1.2. Rationale for the Current Research

To the best of the authors’ knowledge, previous studies have not applied multimodal VLMs to automate triage of endodontic treatment needs from radiographic images containing common acquisition artefacts. Exploration into the topic is especially relevant in resource‐constrained settings where diagnostic decision‐making is challenged by time pressure, limited equipment and inconsistent imaging quality, especially when AI‐based radiographic tools have been demonstrated to enhance diagnostic accuracy among dental students, thereby extending their utility beyond clinical practice into preclinical education and clinical training [8]. Multimodal chatbots can support time‐efficient, contextual clinical reasoning during placements without substantially interrupting clinical duties. It does, however, risk issuing misinformation from inaccurate data sources that are highly cited and remain a factor in the consideration of further investigation [9]. In light of the current global context and shift towards diagnostic AI, the current study aims to evaluate the diagnostic potential of multimodal VLMs for use in real‐world hospital emergency dental settings where rapid endodontic triage can be problematic for numerous reasons, where it is essential to accurately and rapidly triage patients presenting with endodontic problems [2, 3].

### 1.3. Study Approach

This study aimed to develop an AI‐assisted dental radiographic diagnostic framework using multimodal VLMs. Four multimodal models were tested that combine radiographic images and relevant textual data from clinical records. BLIP, CLIP, Florence 2 and PaLIGemma were fine‐tuned using quantisation‐aware training (Llava 13B model) and low‐rank adaptation (LoRA) techniques to make them capable of operating under constrained resources.

The current investigation saw to implement these models under a hospital‐based setting with endodontic radiographs affected by common errors such as cone‐cutting, elongation, foreshortening, misalignment and missing apices. The evaluation metrics used focused on linguistic accuracy and clarity rather than domain‐specific terminology, as current multimodal VLMs are generally trained on diverse, non‐specialist global datasets  [10].

### 1.4. Research Questions

Based on the proposed approach, the following research questions were posed:

1. Can quantisation‐aware training reduce computational costs for multimodal AI without compromising accuracy in triaging endodontic cases?
2. How do different multimodal VLMs compare in performance when trained and evaluated on dental radiographic datasets?
3. Are VLMs capable of generating clinically relevant captions for various stages of endodontic treatment?
4. What performance differences exist between baseline and quantisation‐optimised multimodal models in interpreting endodontic radiographs?

## 2. Materials and Methods

The multicentre research adhered to the Checklist for Artificial Intelligence in Medical Imaging (CLAIM) and maintained compliance with PEP‐8 programming standards to ensure structured computational implementations [11]. This study was approved by Square Hospital Bangladesh and governed under the Institutional Review Board of North South University (2023/OR‐NSU/IRB/0503) to access on‐site data. Deidentified data sharing, transfer, subsequent analyses and collaborative approaches were included in the ethical approval from the University of Adelaide Human Research Ethics Committee (HREC‐2023‐073). The study was partially supported by the University of Adelaide Kwok Lee Bequest (350‐75134777). The funding body had no role in study design, data collection, analysis, interpretation or the decision to publish. All research procedures adhered to the STROBE guidelines for reporting observational studies [12].

### 2.1. Research Design

Raw dental images and corresponding clinical notes were collected from a hospital dental outpatient department and several emergency dental services.

The dataset comprised 4276 dental radiographs, including 3360 orthopantomograms and 916 radiovisiographs, obtained across a 1‐year period. The radiographs were subclassified into three stages of endodontic treatment: need endodontic treatment, incomplete endodontic treatment and complete endodontic treatment. A minimum resolution threshold of 20 lp/mm was the baseline for acceptability in the current AI‐based diagnostic accuracy [13]. 676 radiographs fell below this threshold and were manually excluded, leaving 3600 radiographs for inclusion in the study.

Annotation and labelling were performed using open‐source software (LabelImg; Tzutalin 2015), under the supervision of registered dentists. The annotators were members of the research team (MFST, MAP and AZA) and were directly guided on the retrospective records by the attending dentists on rotation. The dentists involved ranged from recent graduates to those with a minimum of 5 years of clinical experience, ensuring a consistent and clinically informed annotation process.

All data were deidentified on site in accordance with protocols approved by the North South University Ethics Committee (2023/OR‐NSU/IRB/0503). The deidentified dataset was subsequently preprocessed, characterised and analysed following methods approved by the University of Adelaide Human Research Ethics Committee (HREC‐2023‐185).

### 2.2. Data Preprocessing

**Figure 1:**
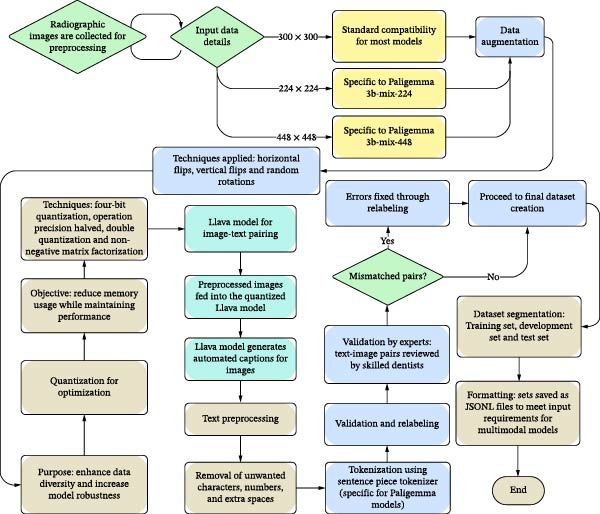
Overview of the workflow for image selection and classification in endodontic analysis.

Figure 1 provides an overview of the workflow used in this study. The radiographic images were enhanced through various augmentation techniques, including horizontal and vertical flipping and random rotations, to increase dataset diversity and improve model generalisability. Prior to augmentation, images were resized to 300 × 300 pixels (for BLIP, CLIP and Florence), with specific dimensions of 224 × 224 and 448 × 448 used for Paligemma model variants [14].

**Figure 2:**
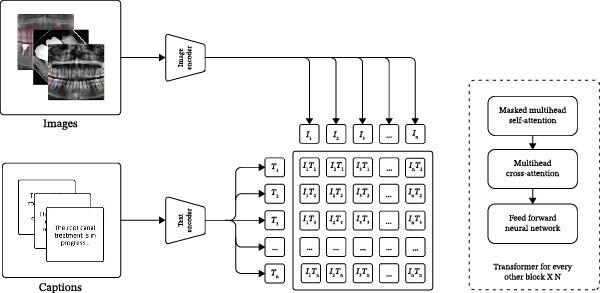
A vision‐language model architecture that integrates an image encoder and a text encoder to process multimodal inputs through some attention mechanisms.

Annotations were generated using a quantised version of the LLaVA‐13B model [15]. ‘Zero‐shot’ machine learning refers to a generative AI model’s ability to perform a task it was never explicitly trained on as it refers to its pre‐learned knowledge. Captions in the current study were produced through zero‐shot prompting and then reviewed alongside clinical notes, and labels refined accordingly. Figure 2 outlines a full end‐to‐end workflow.

Quantisation‐aware training [16] was applied to the LLaVA‐13B model, and the labelled dataset was split into training, validation and test groups. Texts associated with each image were preprocessed using tokenisation [17] to remove irrelevant characters, spaces and numerals. Labels requiring correction were reprocessed through the LLaVA model and were followed by revalidation by clinical experts.

All datasets were saved in JSONL format. Supporing Information 1 illustrates the preprocessing pipeline that includes artefact and noise removal, background cropping, image resizing and conversion from .tif to .png image format. Radiographic images were then organised and fed back into the LLaVA downstream.

### 2.3. Dataset Categorisation

The dataset was classified into three categories with a detailed breakdown of the sample size tied to each category provided in Supporting information 2. The classification was as follows:

1. Need for endodontic treatment: radiographs that demonstrated carious lesions extending into the pulp and clinically confirmed irreversible pulpitis. These teeth would not have prior endodontic intervention.
2. Incomplete endodontic treatment: radiographs in which treatment was perhaps started but not completed. This class was designed to be versatile and could include radiographs showing pulp extirpation, partially instrumented canals and root canal fillings with gutta‐percha. Alternatively, this class could exhibit sealers exhibiting unacceptable length, density or taper or canals prepared and medicated with radiopaque intracanal materials or pre‐sealed substances without definitive coronal restoration.
3. Complete endodontic treatment: radiographs with evidence of fully obturated canals and associated clinical notes to confirm that the patient was likely clinically asymptomatic upon successful follow‐up outcomes, with or without information of referral in the clinical notes for the provision of definitive restoration in a private practice.

### 2.4. Dataset Optimisation

Figure 2 outlines the VLM used in this study. Image‐caption pairs were generated using the quantised LLaVA model [18], with each image processed through a dedicated image encoder to extract salient visual features. This was followed by a text encoder which preserved the semantic and contextual relationships but converted the associated captions into numerical vectors. The input data was pushed through the feed‐forward layers to generate textual outputs and then compared against the corresponding clinical notes [19].

LLaVA model underwent double quantisation followed by non‐negative matrix factorisation [20]. Preprocessed radiographic images were fed into the quantised LLaVA model for zero‐shot prompting to create image‐to‐text pairs [21].

Tokenization for the Paligemma model was performed using a ‘sentence‐piece tokenizer’ [22]. If discrepancies were identified between generated captions and clinical notes, practitioners were consulted for corrections, and relabelling was conducted as needed. Following expert validation, the finalised dataset was split into training, development and testing subsets.

### 2.5. Evaluation Metrics

The multimodal models were quantitatively evaluated using the following metrics:

1. Bilingual Evaluation Understudy (BLEU): measures the overlap of words or short phrases (n‐grams) between the generated output and the reference text.
2. Recall‐Oriented Understudy for Gisting Evaluation (ROUGE): evaluates recall by identifying overlapping units such as n‐grams and word sequences between the generated and reference text.
3. Metric for Evaluation of Translation with Explicit ORdering (METEOR): assesses word‐level matches between the candidate and reference text, accounting for paraphrasing, stemming and synonymy.
4. Consensus‐based Image Description Evaluation (CIDEr): mmeasures the consensus between a generated caption and a set of human references, using term frequency–inverse document frequency (TF‐IDF) weighting to emphasise unique or contextually important words. CIDEr was assessed alongside METEOR to evaluate paraphrasing capability.
5. Loss trends and convergence: used to monitor whether the model’s performance is improving (indicated by decreasing loss) and to determine the optimal point to stop training to prevent overfitting or inefficient computation.

## 3. Results

### 3.1. Hyper Parameter Tuning

A four‐bit quantisation scheme was incorporated to optimise the model size without any loss in accuracy, which was particularly important in the Llava 13 b model. This ensured sustained performance while enhancing memory usage.

Hyperparameters were defined (Supporting information 3) by applying the rank to 16 in BLIP and CLIP, applying a scaling factor of 32 and using 5% dropout results in complexity reductions yet sustains performance. Another example entails different configurations for the different Florence variants, given their larger parameter size: rank 8, scaling factor 8 and a dropout rate of 50%, with bias not altered. The tested parameters offered a well‐balanced memory efficiency with generative accuracy. Of note, horizontal flipping, rotation and resizing improved BLEU‐4 scores by ~20% along with the results in the ROUGE, METEOR and CIDEr scoring metrics.

### 3.2. Quantitative Performance Metrics Following Optimisation

The evaluation of multimodal models using BLEU, ROUGE, METEOR and CIDEr metrics is documented in Supporting information 4. The BLIP base and large variants both initially scored 0.9113 (BLEU‐1) and 0.8148 (BLEU‐4), but fine‐tuned optimisation improved scores to ~99%. BLEU‐4 scores on CLIP models improved from 0.4855 to 0.7896. For CLIP base, unigram scores rose from 0.6251 to 0.8107. Florence 2 variants saw BLEU‐1 improve from 0.3625 to 0.5686 and BLEU‐4 from 0.1547 to 0.2558. Paligemma models (3b‐mix‐224 and 3b‐mix‐448) demonstrated BLEU‐1 scores improving from 0.4747 and 0.4821 to 0.6263 and 0.6613, respectively. BLEU‐4 scores similarly rose from 0.2659 and 0.2741 to 0.3499 and 0.3907. Only the large Florence 2 model performed poorer compared to its base counterpart.

BLIP models scored 0.9916 on ROGUE‐1 and 0.9801 on ROUGE‐2. CLIP models found ROUGE‐1 improving from 0.8909 to 0.9042. Paligemma variants 3b‐mix‐224 rose from 0.5589 to 0.6171 (ROUGE‐1), and 3b‐mix‐448 from 0.5730 to 0.6542. Both versions also saw notable ROUGE‐2 and ROUGE‐L gains, with ROUGE‐L increasing to 0.6320 in the 448 model. Florence 2 models demonstrated significant boosts, with the base model increasing ROUGE‐1 from 0.5118 to 0.5650 and ROUGE‐L from 0.4013 to 0.5053.

The BLIP model’s METEOR score following optimisation increased from 0.5846 to 0.7454, and CIDEr from 5.4964 to 9.6759. CLIP models improved their METEOR and CIDEr scores to 0.7033 and 4.8435, respectively. Florence 2’s base CIDEr score rose from 0.3481 to 0.5747, and the large model saw a jump from 0.0659 to 0.5191. Paligemma’s CIDEr scores doubled, with the 448 variant improving from 0.7624 to 1.7558.

### 3.3. Qualitative Evaluation of Loss Trends and Convergence

Base models typically begin with limited pretraining and are expected to show a sharp decline in the loss curve as they begin learning from the data and adapting to the task. In contrast, large models are heavily pretrained and already contain extensive prior knowledge. As a result, they are better equipped to filter out irrelevant information and retain only the knowledge necessary for the specific task, allowing them to make more informed decisions from the outset.

Figure 3A demonstrates the training loss curves over 100 epochs for both the base and large versions of the BLIP model. The baseline performance of the base model showed a sharp decline in training loss during the initial epochs, followed by a noticeable change in learning dynamics, as seen by an increase in metric output after epoch 50. The optimised model had training loss plateaued after 20 epochs. Optimisation supported the models to reach stability by epoch 30, although it did not outperform the baseline in final loss. The corresponding training loss curves are illustrated in Figure 3B.

Figure 3Comparison of training loss curves for base and large BLIP models: (A) shows the training loss curves for the baseline (left) and optimisation methods (right) of the base model, respectively. (B) Illustrate the training loss curves for the baseline (left) and optimisation methods (right) of the large model, respectively.

**Figure:**
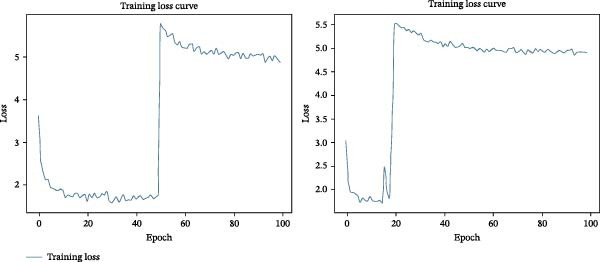
(A)

**Figure:**
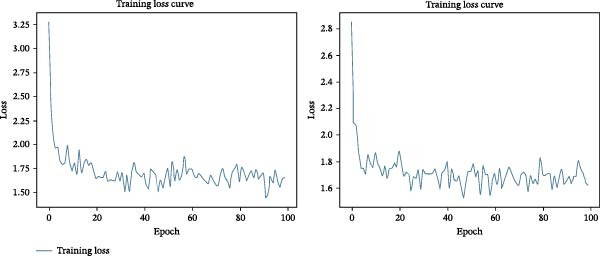
(B)

### 3.4. Generative Captions

An interobserver agreement assessment across the clinical records and corresponding images demonstrated a 92% agreement rate (Cohen’s *κ* = 0.87), indicating strong clinical relevance when relying solely on image‐based inputs. While the models generally performed well in basic endodontic triage, several key considerations and areas for improvement were identified. Table 1 presents selected triage outcomes with corresponding imaging data and clinical notes to facilitate a qualitative assessment of model performance.

**Table 1:** Qualitative analysis of generated captions for endodontic triage.

Radiographic image	Radiographic error	Clinical note caption	Generated caption
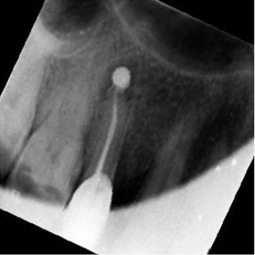	Round cone cut, tilted occlusal plane	Root canal treatment complete, canal oversealed, definitive restoration placed.	The root canal treatment is complete. Limitation: lacks procedural specifics.
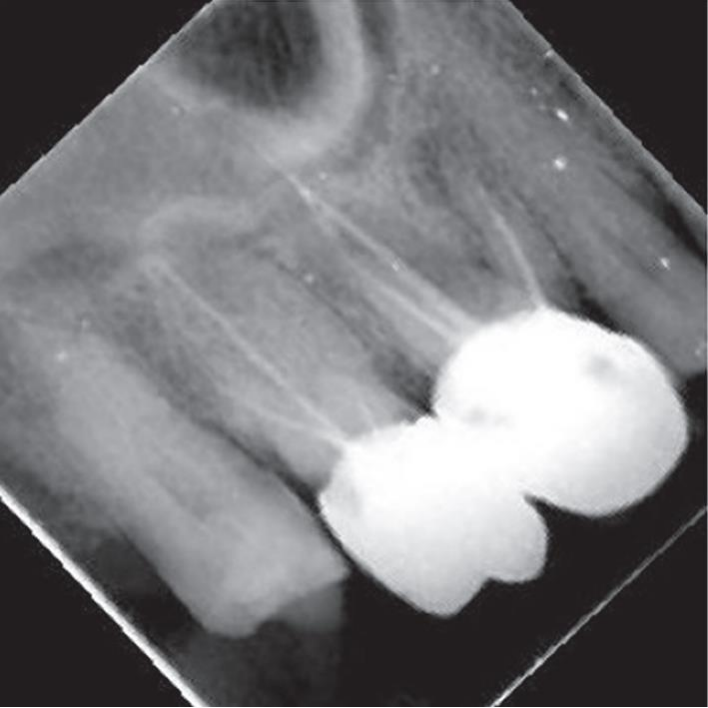	Tilted occlusal plane, misalignment	Completed root canal treatment in teeth #25 and #26 with incomplete root canal treatment (i.e., canal obturation, classified as root canal fillings with gutta‐percha and sealer exhibiting unacceptable length, density, or taper.)	The image sections show varying stages of root canal treatment completion, from incomplete to fully completed procedures. Limitation: lacks technical assessment details.
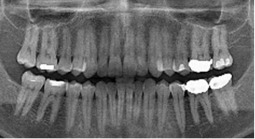	Patient position too far back	Tooth 26 has incomplete endodontic treatment with crown placement, teeth 36, 37 have no endodontic treatment with full coverage crown and secondary caries forming with possible involvement to the pulp.	The right section of the image shows teeth with incomplete root canal treatment, requiring further intervention. Limitation: lacks specificity regarding treatment progression.
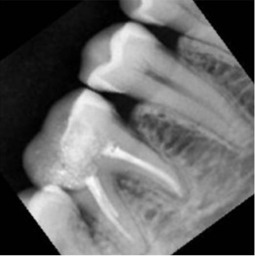	Incomplete image at apex, misalignment	Completed endodontic treatment of tooth 46 with a calcified distolingual canal and an underextended seal; the tooth is asymptomatic with no remarkable clinical findings.	The image shows a tooth with a completed root canal treatment, but the surrounding teeth need further intervention. Limitation: inaccurate assumption about neighbouring teeth condition; contradicts overall healthy ground truth.

## 4. Discussion

Historically, deep learning has led the forefront of image analysis and lesion segmentation from heterogenous endodontic sources that were challenging to process through manual intervention [23, 24]. Global research has now demonstrated that deep‐learning‐based segmentation and classification can rival diagnostic proficiencies of specialists in identifying apical periodontitis, resorption, vertical root fractures, root canal morphology, working length and apical foramen location while demonstrating promise in predicting retreatment outcomes, especially relevant if considering the complex decision‐making that goes behind determining the appropriate management of the clinically significant proportion of failed endodontic cases [25].

Nevertheless, diagnostic performance does vary across imaging modalities with the introduction of complexities within the dentition, such as restored versus obturated and restored tooth conditions. In such instances, trained models experience substantial loss in sensitivity owing to image quality and annotation standards [25, 26]. Many such AI systems are unimodal and are constrained by uncorrectable heterogeneous methodologies of retrospective data, affecting generalisability and increasing cognitive bias [23]. In such instances, conditions such as vertical root fractures on previously endodontically treated teeth remain diagnostically challenging [26–28]. Finally, it is no surprise that predictive systems that operate on predefined parameters are most limited by the quality and completeness of clinical records being input, which are themselves highly variable across clinicians.

In summary, unimodal reasoning systems for endodontics, or dental for that matter of fact, rely on similarity to prior cases, limiting performance when complex cases arise, making the models error susceptible. This is undesirable in a hospital setting where almost all referred cases pose some form of complexity. Training a unimodal model on retrospective or institution‐specific datasets might reduce generalisability thereby making the approach both impractical and unfeasible [27].

The current study aimed to extend beyond previous research by attempting to transition from a unimodal, error‐susceptible assessment towards a multimodal, error‐tolerant system for endodontic triage. While the generated captions in the current assessment demonstrated a reasonable understanding of image content, they frequently failed to convey the key information, such as the status of root canal treatment, the likely treatment done, or the condition of the adjacent teeth in full, thereby reducing clinical utility. In some cases, there were substantial inconsistencies between the generated captions and the ground truth.

BLIP variant models were seen to outperform the other models in the current study. These models were trained using the AdamW optimiser with a categorical cross‐entropy loss function that could theoretically explain some of the success BLIP achieved. This approach was different from that of CLIP variants. CLIP lacked a decoder component in their architecture and instead relied on normalised text and image embeddings for similarity‐based caption generation. Paligemma and Florence‐2 variants were fine‐tuned with complex mechanics such as learning rate schedulers, warm‐up strategies to stabilise training and Paligemma variants were trained using stochastic gradient descent (SGD) optimisers. BLIP did not possess any of these advanced approaches and yet, interestingly, performed better.

The primary limitation in the generative text identified was the underreporting of condition severity or technical nuances in the available documentation. However, by reducing computational precision from 32‐bit to four‐bit processing, which is an 87.5% decrease in computational load, there is potential to incorporate additional analytical steps. This allows for further refinement of the triage process without compromising system efficiency. Furthermore, training loss analyses suggest economically efficient parameter storage and parameter loading make quantisation a good feature for a resource‐constrained environment [29].

To put the significance of the current research into perspective, a deeper dive is required into earlier studies. Studies such as Hasan et al. [30] focused on image denoising and class balancing using CNNs with 2,226 images to improve diagnostic accuracy, while Moidu et al. [4] used YOLOv3 for periapical lesion scoring, limited to intraoral radiographs only. Ozbay et al. [3] concentrated on endodontic fragment detection using Mask R‐CNN, and Wang et al. [5] explored neural networks for root canal segmentation on CBCT data. Ji et al. [7] demonstrated the utility of BLIP for clinical captioning, although not applied to dental imaging, and Qu et al. [31] focused on microsurgery outcome prediction using machine learning on surgical datasets. Unlike these single‐task or narrow‐domain approaches, the current study implemented and optimised multiple VLMs (BLIP, CLIP, Florence 2 and Paligemma) for endodontic triage, employing quantisation‐aware training, data augmentation and human‐verified captions to adapt and expand on their respective methodologies. This approach addressed the small sample sizes, lack of domain adaptation and reliance on a single imaging modality.

The current study presented some mathematical complexities owing to the heterogeneity of the dataset, the use of quantisation‐aware training, and the inherent diversity of multimodal models tested. First, resizing a large dataset of images to different resolutions complicated image processing. The dataset of 3600 images had to be resized to multiple resolutions (224 × 224, 256 × 256 and 512 × 512) to ensure compatibility with various model architectures. This introduced a computational complexity that scaled with the product of image count and resolution size, or *O*(*n* · *r*), where higher resolutions increased processing load.

Next, quantisation‐aware training was implemented using four‐bit quantisation, reducing memory requirements by 87.5% from 32‐bit floating‐points to four‐bit integers. While beneficial to reduce memory load, it made it necessary for gradient approximation during the fine‐tuning workflow in low‐bit environments. Finally, the training and evaluation of four distinct multimodal models (BLIP, CLIP, Florence 2 and Paligemma) required over 50 trials per model for hyperparameter tuning. Conducting such extensive optimisation in a clinical setting would demand substantial GPU resources and prolonged runtime, undermining the goal of low‐maintenance, generalisable multimodal models. Further research is needed to develop efficient strategies for training and fine‐tuning pretrained models to specific clinical cohorts and to evaluate their practical performance in real‐world clinical environments.

As of 2025, the generated captions can generally identify the overall completion status of root canal treatments but lack procedural, technical and contextual specificity, limiting their reliability for precise clinical interpretation and assessment of treatment progression until more robust, clinically validated models become publicly available.

## 5. Conclusion

The following conclusions were drawn from the current research:

1. Quantisation reduced memory consumption by 87.5% while preserving diagnostic accuracy within a 0.5% error margin. It makes multimodal triage clinically feasible with minimal computational demands without compromising performance.
2. BLIP surpassed all models across BLEU, ROUGE, METEOR and CIDEr, while CLIP showed notable improvement post‐optimisation. Florence 2 and PaLIGemma withered slightly but still benefited from quantisation and augmentation.
3. Models like BLIP, CLIP, Florence 2 and PaLIGemma, proceeded by quantised Llava‐13 B, generated 92% assessments to practitioner‐directed triage.
4. Quantisation‐aware optimisation improved BLEU‐4 by at least 17.3%, METEOR by at least 11.1%, ROUGE‐L by at least 9.8% and CIDEr by at least 75.5% across all models.

## Author Contributions

Conceptualisation, formal analysis, methodology: Md Fahim Shahoriar Titu, Mahir Afser Pavel, Afifa Zain Apurba, Saif Ahmed, Shafin Rahman, James Dudley and Taseef Hasan Farook. Data curation, investigation: Md Fahim Shahoriar Titu, Mahir Afser Pavel and Afifa Zain Apurba. Funding acquisition: James Dudley and Taseef Hasan Farook. Project administration, resources, validation: Saif Ahmed, Shafin Rahman, James Dudley and Taseef Hasan Farook. Visualisation: Md Fahim Shahoriar Titu, Mahir Afser Pavel, Afifa Zain Apurba and Saif Ahmed. Roles/writing – original draft: Md Fahim Shahoriar Titu, Mahir Afser Pavel, Afifa Zain Apurba, Saif Ahmed and Shafin Rahman. Writing – review and editing: Saif Ahmed, James Dudley and Taseef Hasan Farook.

## Acknowledgments

Manuscript grammar was revised with the help of generative AI (ChatGPT).

## Funding

This study was partially supported by the University of Adelaide Kwok Paul Lee Bequest (Grant 350‐75134777). Open access publishing facilitated by Adelaide University, as part of the Wiley ‐ Adelaide University agreement via the Council of Australian University Librarians.

## Disclosure

The funding body had no role in the study design, data collection, analysis, interpretation or in the decision to publish the findings.

## Conflicts of Interest

The authors declare no conflicts of interest.

## Supporting Information

Additional supporting information can be found online in the Supporting Information section.

## Supporting information


**Supporting Information 1** An infographic illustration of the proposed framework and preprocessing workflow applied in developing the current multimodal system.

**Supporting Information 2** Distribution of Radiographic Images Across Endodontic Classifications and Selection Criteria.

**Supporting Information 3** Information on hyperparameter tuning.

**Supporting Information 4** Performance metrics of multimodal systems evaluated using BLEU, ROUGE, METEOR, and CIDEr scores.

## Data Availability

The codes and scripts for the current research are available on GitHub; https://github.com/MdFahimShahoriar/Endodontic-using-VLM (last accessed on 16 January 2025).
