# Supplementary material for: Error‐Tolerant Multimodal Vision‐Language Models for Endodontic Triaging: A Cross‐Sectional Study
Source: Int J Dent. 2026 Jan 31;2026:4148741. doi: 10.1155/ijod/4148741 (PMC12860215; doi:10.1155/ijod/4148741)

Supplementary File 1. Proposed framework and preprocessing workflow

The figure below illustrates the proposed workflow, highlighting the distinctions between this approach and conventional single-modality CNN or object detection models (e.g., YOLO).

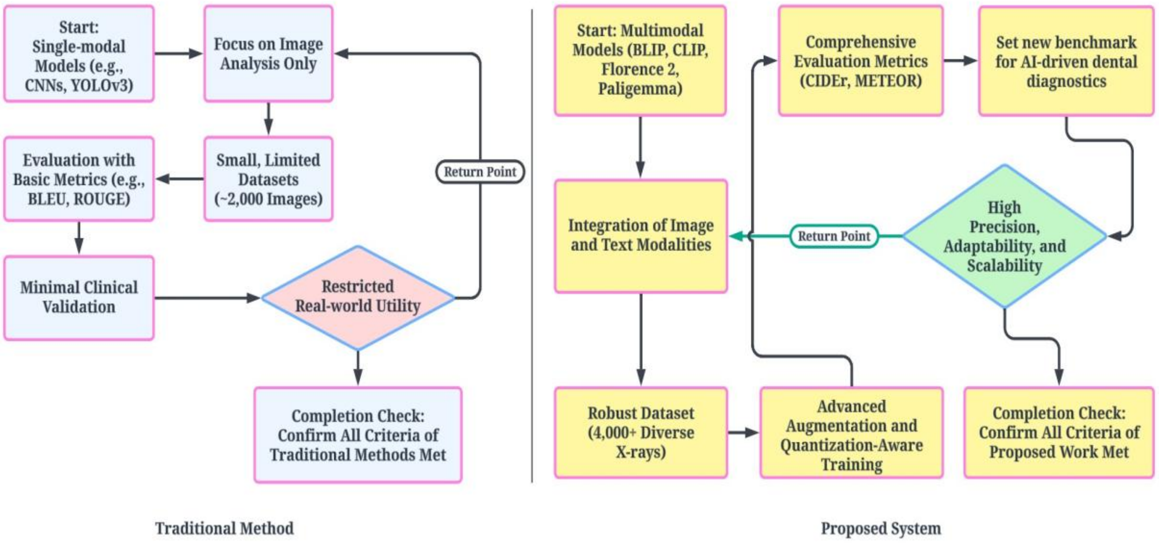

The Figure below illustrates a summarised visualisation of the preprocessing workflow for the current study

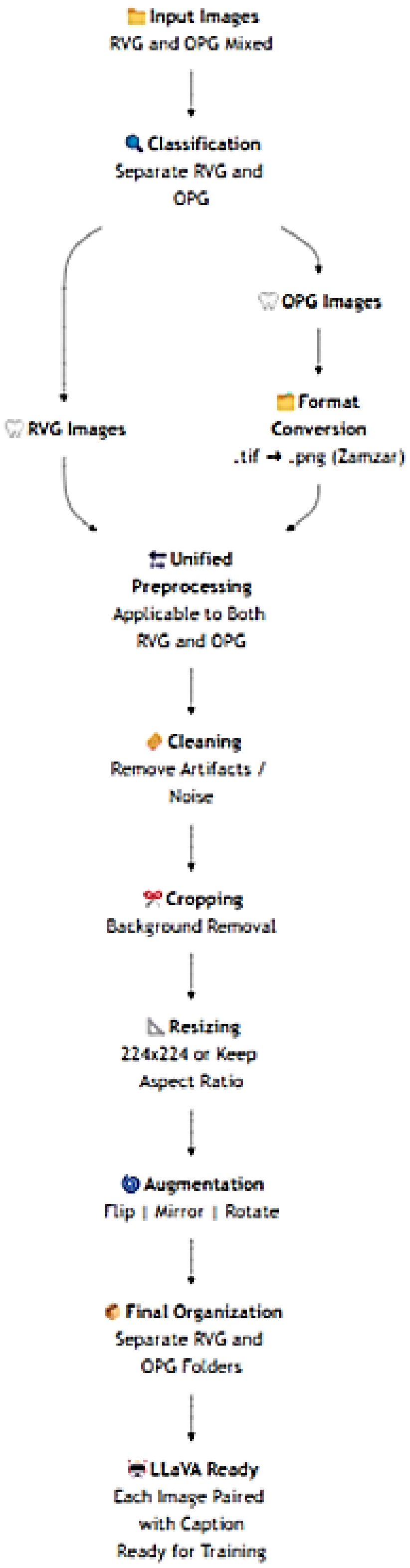

Supplement: Supplementary file 1 — Supporting Information 1 An infographic illustration of the proposed framework and preprocessing workflow applied in developing the current multimodal system. [file IJOD-2026-4148741-s001.pdf]
