# Supplementary material for: Error‐Tolerant Multimodal Vision‐Language Models for Endodontic Triaging: A Cross‐Sectional Study
Source: Int J Dent. 2026 Jan 31;2026:4148741. doi: 10.1155/ijod/4148741 (PMC12860215; doi:10.1155/ijod/4148741)
Supplement: Supplementary file 2 — Supporting Information 2 Distribution of Radiographic Images Across Endodontic Classifications and Selection Criteria. [file IJOD-2026-4148741-s002.pdf]

Supplementary Table 2: Distribution of Radiographic Images Across Endodontic Classifications and Selection Criteria

Type Of Images	Usable Total	After manual selection	Class	Images per class
RVG	916	900	Complete Endodontic Treatment	300
			Incomplete Endodontic Treatment	290
			Need Endodontic Treatment	310
OPG	3360	2700	Complete Endodontic Treatment	870
			Incomplete Endodontic Treatment	925
			Need Endodontic Treatment	905
Total	4276	3600	Class = 03	3600

RVG = RadioVisioGraphy (Intraoral Radiograph), OPG = Orthopantomogram (full mouth Radiograph)
