# Supplementary material for: Error‐Tolerant Multimodal Vision‐Language Models for Endodontic Triaging: A Cross‐Sectional Study
Source: Int J Dent. 2026 Jan 31;2026:4148741. doi: 10.1155/ijod/4148741 (PMC12860215; doi:10.1155/ijod/4148741)
Supplement: Supplementary file 3 — Supporting Information 3 Information on hyperparameter tuning. [file IJOD-2026-4148741-s003.pdf]

Supplementary Table 3. Hyperparameter tuning

Model	BS	Opt.	LR	Loss Fn.	Duration	Sched.	WU
BLIP-B	2	AdamW	1e-3	CCE	100e	None	–
BLIP-L	2	AdamW	1e-3	CCE	100e	None	–
CLIP-B	2	AdamW	1e-3	CLIP	100e	None	–
CLIP-L	2	AdamW	1e-3	CLIP	100e	None	–
Florence-2B	2	AdamW	1e-3	CCE	100e	Linear	–
Florence-2L	2	AdamW	1e-3	CCE	100e	Linear	–
PaLM-3b-224	2	SGD	1e-3	CCE	8ks	Cosine	10%
PaLM-3b-448	2	SGD	1e-3	CCE	8ks	Cosine	10%

BS = Batch Size, Opt. = Optimizer, LR = Learning Rate, CCE = Categorical Cross Entropy, e = epochs, ks = thousand steps, WU = Warmup, B = Base, L = Large

The hyperparameters modified include batch size, optimizer type, learning rate, loss function, training duration (epochs or steps), learning rate scheduler, and warm-up percentage. The batch size was set to 2 to allow for stable training but increased computational demands. AdamW was employed for its efficiency and stability during training, while SGD was used for its generalization capability.

The learning rate was set to 0.001, balancing convergence speed and training stability. Loss functions included Categorical Cross-Entropy for classification tasks and a custom CLIP loss for contrastive learning tasks in multimodal Image-Media models. Training duration was defined by either 100 epochs or 8000 steps, allowing sufficient learning time without excessive computational overhead.

The linear and cosine learning rate scheduling strategies were applied to enhance generalisation and reduce overfitting. A warm-up percentage was finally incorporated to

gradually increase the learning rate at the start of training, helping to avoid instability and divergence.
