# Supplementary material for: Error‐Tolerant Multimodal Vision‐Language Models for Endodontic Triaging: A Cross‐Sectional Study
Source: Int J Dent. 2026 Jan 31;2026:4148741. doi: 10.1155/ijod/4148741 (PMC12860215; doi:10.1155/ijod/4148741)
Supplement: Supplementary file 4 — Supporting Information 4 Performance metrics of multimodal systems evaluated using BLEU, ROUGE, METEOR, and CIDEr scores. [file IJOD-2026-4148741-s004.pdf]

Supplementary File 4

Table 1: BLEU Scores for Vision language models

Methods	Models	BLEU-1	BLEU-2	BLEU-3	BLEU-4
**Baseline**	BLIP BASE	0.9113	0.8792	0.8472	0.8148
	BLIP LARGE	0.9113	0.8792	0.8472	0.8148
	CLIP BASE	0.6251	0.5764	0.5300	0.4855
	CLIP LARGE	0.6251	0.5764	0.5300	0.4855
	FLORENCE-2 BASE	0.3625	0.2521	0.1937	0.1547
	FLORENCE-2 LARGE	0.2151	0.1726	0.1359	0.1080
	PALIGEMMA 3B-MIX-224	0.4747	0.3764	0.3093	0.2659
	PALIGEMMA 3B-MIX-448	0.4821	0.3857	0.3191	0.2741
**Optimization**	BLIP BASE	0.9917	0.9877	0.9835	0.9805
	BLIP LARGE	0.9917	0.9877	0.9835	0.9805
	CLIP BASE	0.8107	0.8038	0.7965	0.7896
	CLIP LARGE	0.8107	0.8038	0.7965	0.7896
	FLORENCE-2 BASE	0.5686	0.4510	0.3385	0.2558
	FLORENCE-2 LARGE	0.5386	0.4145	0.3067	0.2231
	PALIGEMMA 3B-MIX-224	0.6263	0.5272	0.4284	0.3499
	PALIGEMMA 3B-MIX-448	0.6613	0.5658	0.4704	0.3907

Note: Baseline = The model’s original performance before any fine-tuning or improvements, Optimization = Enhanced performance after applying fine-tuning or task-specific improvements.

Table 2: ROUGE scores for Vision language models

Methods	Models	ROUGE-1	ROUGE-2	ROUGE-L
	BLIP BASE	1.0000	1.0000	1.0000
	BLIP LARGE	1.0000	1.0000	1.0000
	CLIP BASE	0.8909	0.8897	0.8909
**Baseline**	CLIP LARGE	0.8909	0.8897	0.8909
	FLORENCE-2 BASE	0.5118	0.2661	0.4013
	FLORENCE-2 LARGE	0.4386	0.2839	0.3801
	PALIGEMMA 3B-MIX-224	0.5589	0.3887	0.4978
	PALIGEMMA 3B-MIX-448	0.5730	0.4062	0.5065
	BLIP BASE	0.9916	0.9801	0.9916
	BLIP LARGE	0.9916	0.9801	0.9916
	CLIP BASE	0.9042	0.8909	0.9040
**Optimization**	CLIP LARGE	0.9042	0.8909	0.9040
	FLORENCE-2 BASE	0.5650	0.3511	0.5053
	FLORENCE-2 LARGE	0.5439	0.3176	0.4903
	PALIGEMMA 3B-MIX-224	0.6171	0.4300	0.5966
	PALIGEMMA 3B-MIX-448	0.6542	0.4738	0.6320

Note: Baseline = The model's original performance before any fine-tuning or improvements, Optimization = Enhanced performance after applying fine-tuning or task-specific improvements.

Table 3: METEOR and CIDEr scores for Vision language models

Methods	Models	METEOR	CIDEr
	BLIP BASE	0.5846	5.4964
	BLIP LARGE	0.5846	5.4964
	CLIP BASE	0.6971	1.1330
**Baseline**	CLIP LARGE	0.6971	1.1330
	FLORENCE - 2 BASE	0.2075	0.3481
	FLORENCE - 2 LARGE	0.1883	0.0659
	PALIGEMMA 3B-MIX-224	0.1772	0.8347
	PALIGEMMA 3B-MIX-448	0.1853	0.7624
	BLIP BASE	0.7454	9.6759
	BLIP LARGE	0.7454	9.6759
	CLIP BASE	0.6703	6.4833
**Optimization**	CLIP LARGE	0.6703	6.4833
	FLORENCE - 2 BASE	0.2930	0.5747
	FLORENCE - 2 LARGE	0.2636	0.5191
	PALIGEMMA 3B-MIX-224	0.3251	1.4659
	PALIGEMMA 3B-MIX-448	0.3499	1.7558

Note: Baseline = The model's original performance before any fine tuning or improvements, Optimization=Enhanced performance after applying fine-tuning or task-specific improvements.
